# Shear Strength of Nano Silica High-Strength Reinforced Concrete Beams

**DOI:** 10.3390/ma15113755

**Published:** 2022-05-24

**Authors:** Mahmoud A. El-Mandouh, Mosbeh R. Kaloop, Jong-Wan Hu, Ahmed S. Abd El-Maula

**Affiliations:** 1 Civil Construction Department, Beni-Suef University, Beni-Suef 62511, Egypt; mahmoudaziz_2008@yahoo.com; 2 Department of Civil and Environmental Engineering, Incheon National University, Incheon 22012, Korea; mosbeh@mans.edu.eg; 3 Incheon Disaster Prevention Research Center, Incheon National University, Incheon 22012, Korea; 4 Public Works Engineering Department, Mansoura University, Mansoura 35516, Egypt; 5 Faculty of Engineering, Benha University, Benha 13511, Egypt; ahmed.alsayed@feng.bu.edu.eg

**Keywords:** reinforced concrete, shear behavior, high-strength concrete, nano silica concrete

## Abstract

In this study, the shear strength of sixteen full-scale over-reinforced concrete beams with and without nano silica (NS), constructed from high-strength concrete (HSC), was investigated both experimentally and analytically. Nano silica was used as a partial replacement for Portland cement. According to the NS ratio, the tested beams were divided into four groups: 0%, 1%, 2%, and 3%. Shear span to effective depth (a/d) ratios of 1.5 and 2.5 were used in each group, and two different stirrups ratios (ρ_v_) were employed as 0% and 0.38%. The shear strength provisions used by some international codes, such as the American Concrete Institute (ACI-2019), the Eurocode 2 (EC-2), and the Egyptian Code (ECP 207), were examined when applied to HSC beams with and without NS. The most important factors to consider were the effect of using NS on the shear span to effective depth (a/d) ratio and the shear strength of the beams with and without stirrups. The experimental results were validated using a nonlinear finite element analysis using the computer program ABAQUS. The experimental results showed that increasing the NS ratio reduced the number of cracks, and increased the cracks spacing, as well as reducing crack width. In specimens without stirrups, these effects were more obvious. A rise in the (a/d) ratio increased the number of cracks along the beam length, notably in the mid-span region. For specimens without stirrups and with an (a/d) of 1.5, raising NS from 0% to 1%, 2%, and 3% increased the ultimate load by 13%, 30%, and 39%, respectively, whereas for specimens with an (a/d) of 2.5, the ultimate load increased with approximately the same increase as that in beams with an (a/d) of 1.5 due to using NS. Additionally, the addition of NS to concrete boosted the contribution of the concrete to the shear strength, as shown by the results of beams without stirrups. For specimens with stirrups and an (a/d) of 1.5, raising NS from 0% to 1%, 2%, and 3% increased the ultimate load by 8%, 21%, and 30%, respectively. Additionally, for specimens with stirrups and an (a/d) of 2.5, the ultimate load increased with approximately the same increase as that in beams with stirrups and an (a/d) of 1.5 due to using NS. The test findings indicate that the shear strength calculated using the equations of the ACI 318-19 is more conservative than EC-2 and ECP 207 for NS concrete beams. The finite element program ABAQUS may be successfully used to predict the shear strength of NS concrete beams.

## 1. Introduction

Nowadays, concrete is considered a widespread worldwide construction material. However, concrete is a brittle material and is distinguished by a very low tensile strength. The use of nano silica (NS) for supplementing cementing materials is a new technology that has been applied to concrete manufacturing to enhance the mechanical properties of hardened concrete [1]. In general, nanomaterials have been utilized in enhancing the performance of concrete due to their compacting microstructure influence and hastening the reaction of cement hydration [2,3]. Currently, NS has gained the attention of reinforced concrete (RC) investigators due to its significant enhancement of mechanical and chemical performance in RC structures. NS is a highly effective pozzolanic material featuring extremely fine particles with a high surface area. In an extensive review [4], an article stated the impact of different nanoparticles on the mechanical properties of high-performance concrete (HPC). It was stated that HPC revised by NS has excellent compressive strength compared with conventional HPC. Additionally, it was recommended that the replacement amount of NS cannot be too high, and an amount smaller than 5% is suggested. Furthermore, for HPC made with NS, the improving developments of tensile and bending strengths are like those of compressive strength. In a similar experimental study, the same findings were recognized [5], where the findings revealed that the implementation of NS improved the HPC compressive strength compared with specimens not including NS. An experimental investigation was conducted to determine the optimal quantity of NS when utilized as a replacement for cement content in concrete using three proportions of NS: 1%, 2%, and 3% [6]. It was concluded that the use of NS in the concrete mixture performs not only as a filler to enhance the microstructure but also as an activator to help the pozzolanic reaction, thus causing the development of the durability and mechanical properties of concrete. Additionally, even a minor amount of NS could increase the concrete compressive strength. It was found that NS mixes gave optimum mechanical properties to hardened concrete by using a percentage of 3% as a replacement of cement content, where the concrete compressive strength increased by 37% at 28 days compared with the control mix. Additionally, by using NS 3%, the tensile and flexural strengths were increased by 51% and 43%, respectively. The impact of applying different types of nanomaterials as replacements for cement on the mechanical properties of high-strength concrete (HSC) has been experimentally investigated [7]. It was concluded that 3% was the best dosage of NS, where the improvement of concrete compressive strength was 21% for the 0% NS concrete. Additionally, at the same percentage of NS, the concrete tensile and flexural strengths were improved by 44% and 23%, respectively. Finally, it was recommended that a superplasticizer be used in concrete mixes to enhance the workability. The durability of concrete produced with NS and microsilica (MS) as cement partial replacement has been experimentally explored [8]. It was observed that replacing cement with 2% NS and 8% MS mixture significantly improved the concrete’s durability, where the concrete sample’s water permeability and chloride ion penetrability were decreased by 38% and 47%, respectively. The structural flexural behavior of reinforced concrete (RC) beams constructed with different NS proportions as a partial replacement by weight of cement was experimentally tested [9]. It was found that the concrete cube compressive strength increased with an increasing NS ratio due to the pozzolanic and filling nature of NS. The findings revealed that the optimum NS ratio was 2% for concrete compressive strength, where the strength was increased by 20% compared with 0% NS specimens. Additionally, RC beams containing NS showed an improvement in the first cracking and ultimate loads and a decrease in the deflection at cracking and ultimate load stages. When compared with the reference beam, RC beams with an NS of 3% experienced a 19% increase in ultimate load and a 23% decrease in ultimate deflection. In a similar experimental study, the flexural behavior of RC beams manufactured with different percentages of NS was investigated [10]. Once more, it was demonstrated that NS concrete beams showed high flexural performance, higher failure load capacity, and enhanced ductility when compared with control beams without NS. Additionally, NS improved the RC beams’ crack performance, decreased the number of cracks, and reduced crack width. During another experimental study, the effect of NS and steel fibers (SF) on the cyclic flexural behavior of lightweight concert beams was investigated [11]. It was found that the NS ratio of 3% had the highest concrete compressive strength and modulus of elasticity, where the increase was 36% and 19%, respectively, compared with the specimens without NS. Furthermore, using NS with SF improved the RC beams’ cyclic flexural and energy dissipation capacities by about 11% and 50%, respectively. This was because NS improved the bond strength between SF and the cement, resulting in improved SF crack resistance. The flexural behavior of beams constructed with NS high-strength concrete and reinforced with glass fiber (GFRP) bars was experimentally examined [12]. It was concluded that concrete beams manufactured with NS had enhanced flexural behavior and decreased flexure cracks, in both number and width. The effect of NS additions on the shear and flexural structural behavior of RC beams has been studied [13]. It was discovered that adding NS to concrete mixes increased the shear and bending capacity of beams. The shear behavior of reinforced concrete beams, where NS was used as an additive material in concrete manufacturing with different proportions, was studied in [14]. The results exhibited that, when the percentage of NS was increased from 0% to 3%, the compressive strength, tensile strength, and modulus of elasticity of concrete were increased by about 31%, 45%, and 13%, respectively. In addition, it was determined that NS enhanced the shear behavior of beams at an NS ratio of 3%, and the cracking shear force and the ultimate shear capacity were increased by about 36% and 25% when compared with reference beams without NS. The effect of an NS and microsilica (MS) mixture on the shear behavior of RC beams has been experimentally studied [15]. An NS and MS mixture was used in producing concrete with various ratios as a partial replacement for cement. The results indicated that, compared with reference concrete samples, the concrete compressive strength was increased by 33% when a mixture of 2% NS and 8% MS was used. Additionally, at the same ratio of NS and MS mixture, the beams’ shear strength was improved by 30% and 59 % for beams with and without steel stirrups, respectively, compared with 0% silica mixture beams. Experimental studies related to the shear strength of NS-reinforced concrete beams are few and limited in the literature, since the implementation of NS in the reinforced concrete structural elements is still a new trend. So, one of the strong points of the current study is to contribute to the literature with experimental and analytical shear behavior of NS-reinforced concrete beams.

In this research, the shear strength of sixteen full-scale over-reinforced concrete beams with and without nano silica (NS), constructed from high-strength concrete (HSC), is investigated both experimentally and analytically. When applied to HSC beams with and without NS, the shear strength provisions used by several international codes, such as the American Concrete Institute (ACI-2019) [16], the Eurocode 2 (EC-2) [17], and the Egyptian Code (ECP 207) [18], were investigated. The experimental results were validated using a nonlinear finite element analysis with the computer program ABAQUS [19].

## 2. Experimental Program

### 2.1. Materials

**Figure 1:**
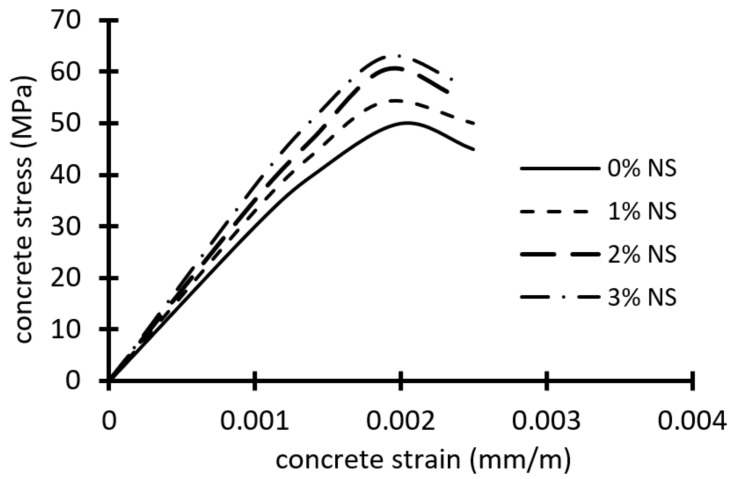
Stress–strain curves for HSC with and without NS.

CEM I 52.50N ordinary Portland cement was utilized. The coarse aggregate was crushed limestone with a size of 8 mm. Natural sand with a fineness modulus of 2.80 was used as the fine aggregate. Nano silica (NS) is an amorphous silica powder that is exceptionally pure and has a large surface area, outstanding adsorption properties, and excellent dispersion. NS is a fine material with a diameter of 50 mm; this makes it 50 times smaller than cement. As a result, adding NS to a lower level of cement replacement improves the mechanical and durability properties of concrete by accelerating the hydration and pozzolanic reaction at an early stage. Table 1 shows the physical and chemical properties of NS. Additionally, a superplasticizer (SP) was employed to secure the regular distribution of the nanoparticles and to provide the workability of the fresh concrete. All four concrete mixes were designed with HSC; the first was without NS, the second was with 1% NS, the third was with 2% NS, and the last was with 3% NS. Table 2 illustrated the mix proportions for four design mixes. The slump, air content, and density tests were used to study the effect of NS on the physical qualities of freshly mixed concrete. It was observed that the addition of NS reduced the slump and air content of the fresh concrete mixture, the concrete mixtures with 2% and 3% NS had the lowest air content. However, the NS had no significant effect on concrete density. The test results on fresh concrete are presented in Table 3. The average cylinder compressive strength fc’ based on three cylinders (φ150 × 300 mm) and cube compressive strength f_cu_ based on three cubes (150 × 150 mm) were determined, as illustrated in Table 4, after 28 days of immersion in a water tank. The average splitting cylinders tensile strengths ft were equal to 3.20 MPa, 3.51 MPa, 3.80 MPa, and 3.90 MPa for mixes without NS, 1% NS, 2% NS, and 3% NS, respectively, as illustrated in Table 2. The results showed that, by increasing NS from 0% to 3%, the concrete compressive strength and tensile strength increased by about 24% and 22%, respectively. In addition, three cylinders were made and tested to evaluate each mix’s stress–strain relationship. The strain of the examined specimens was determined using three strain gages. On each cylinder, strain gauges were linked at the midpoint and at a 120° angle in the horizontal direction. Figure 1 shows the average stress–strain curves for HSC mixtures with and without NS. Increasing the NS ratio has an important impact on the stress–strain curve’s ascending branch.

### 2.2. Description of the Tested Specimens

**Figure 2:**
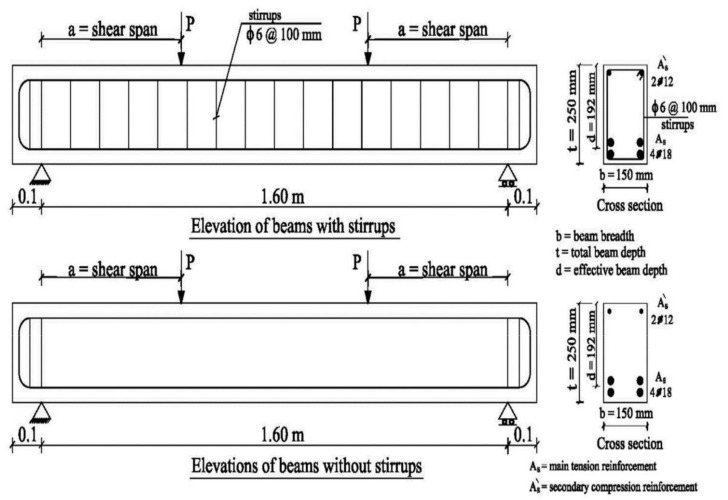
Details of the tested beams.

**Figure 3:**
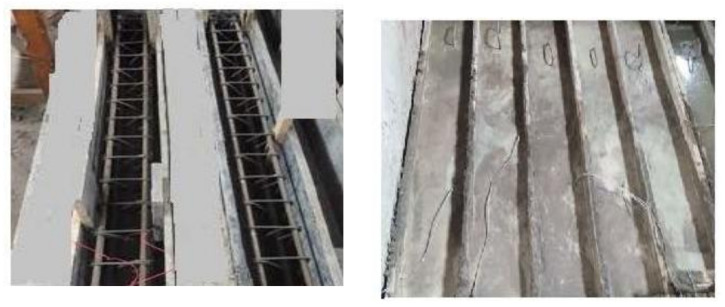
Reinforcement details and casting of some studied beams.

Sixteen simply supported HSC beams with different percentages of NS were experimentally examined up to failure under two-point symmetric loading. All the tested beams had concrete sections of 150 × 250 mm with lengths of 1600 mm. The reinforcement details and concrete dimensions of the tested beams are illustrated in Table 4 and Figure 2. According to the NS ratio, the tested beams were divided into four groups: 0%, 1%, 2%, and 3%. Shear span to effective depth (a/d) ratios of 1.5 and 2.5 were used in each group, and two different stirrups ratios (ρ_v_) were employed at 0% and 0.38%. The stirrups were constructed from 6 mm-diameter bars with 200 mm and 100 mm spacing. For all the tested beams, the main bottom reinforcement was over-reinforced with four bars with a diameter of 18 mm to avoid flexural failure and two bars with a diameter of 12 mm as a top reinforcement. The flexural reinforcement ratio (ρ_L_) for all tested beams was equal to 3.53%. The design yield stress was 410 MPa for longitudinal bars and 243 MPa for stirrups. Longitudinal bars had a design yield stress (*f_y_*) of 410 MPa, whereas stirrups had yield stress (*f_ys_*) of 243 MPa. All specimens were cast and cured with purified water for 28 days before testing. Figure 3 shows the reinforcement details and casting of some tested beams.

### 2.3. Test Setup and Instruments

**Figure 4:**
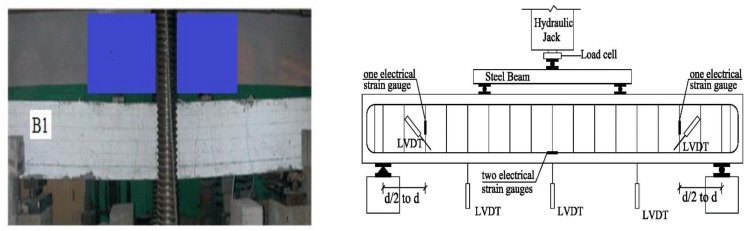
Test setup.

A conventional universal machine was used to test all beams. A load cell with a capacity of 300 kN and an accuracy of 0.1 kN was used to measure the loads. The beams were tested under two-point loads with two shear span–depth ratios of 1.50 and 2.5, respectively. Figure 4 depicts the beams’ test setup and the locations of the LVDTs and the electrical strain gauges. The LVDTs and the electrical strain gauges were symmetrically arranged. A roller and hinged supports were used on the right and left edges of the studied beams, respectively. Two 5 mm-thick steel sheets were put under the loading rods and over the supports to prevent local failure under load. The mid-span deflection of the beams was measured using LVDT. The stirrups strain at the shear span’s midpoint was measured using electrical strain gauges. Additionally, LVDT was used to measure the shear crack widths at the shear span.

## 3. Experimental Results

For all tested beams, Table 5 shows the measured cracking load (P_cr_), ultimate load (P_u_), and mid-span deflection (∆_u_).

### 3.1. Crack Pattern

**Figure 5:**
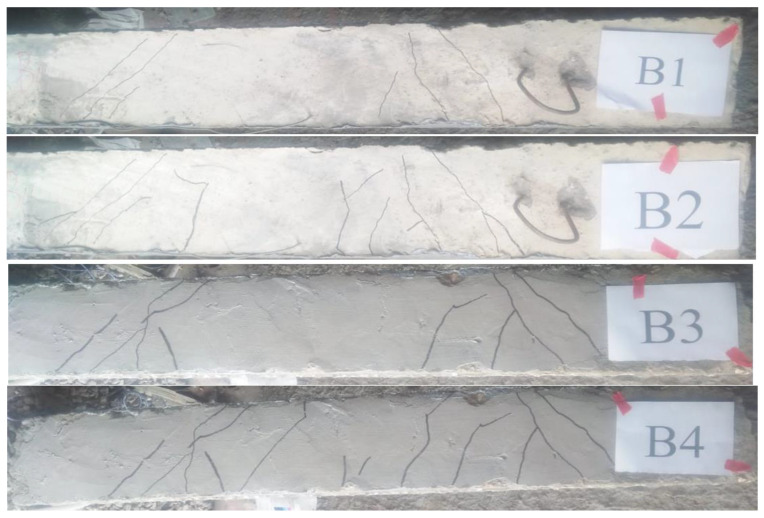

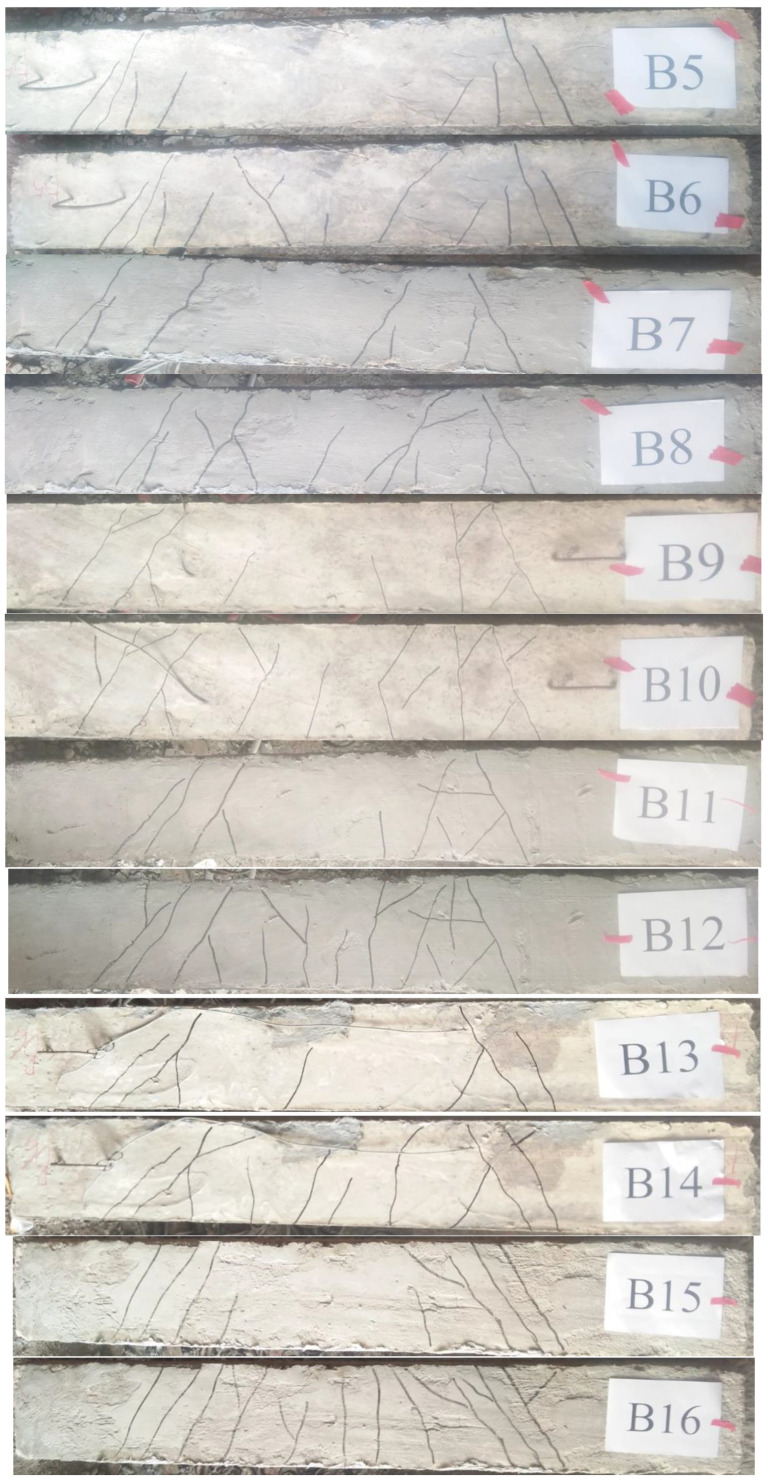
The tested beams’ crack patterns.

Figure 5 shows the schematic cracking patterns of the tested beams at failure. As expected, the tested beams failed in shear. The shear failure was brittle and occurred quickly for beams without stirrups. Generally, for beams with stirrups, the initial crack formed vertically in the central region with some fine vertical cracks appearing at the mid-span sections. On the other hand, for beams without stirrups, the initial crack was not formed in the central region. As the load was increased, inclined cracks formed between the load and the support points in the shear span region, which then spread towards the supports due to the increased inclination. Induced inclined cracks propagated and spread as the load gradually increased. At failure, one of these diagonal cracks suddenly expanded into the compression zone and down to the supporting region, causing the beams to break in shear. Generally, for beams with stirrups and with (a/d) of 2.5, the number of cracks was almost larger than the corresponding beams with (a/d) of 1.5. For specimens without stirrups and having (a/d) of 1.5, raising NS from 0% to 1%, 2%, and 3% increased the cracking load by 15%, 30%, and 33%, respectively, whereas for specimens without stirrups and having (a/d) of 2.5, the cracking load increased by 20%, 27%, and 34%, respectively. According to the findings from increasing NS from 2% to 3%, the cracking load was slightly enhanced. For specimens with stirrups and having (a/d) of 1.5, raising NS from 0% to 1%, 2%, and 3% increased the cracking load by 4%, 26%, and 36%, respectively, whereas for specimens with stirrups and having (a/d) of 2.5, the cracking load increased by 20%, 49%, and 54%, respectively. As a result, the stirrups were more effective for beams with a greater NS content. The results showed that increasing the NS ratio reduced the number of cracks, and increased the cracks spacing, as well as reducing the crack width. In specimens without stirrups, these effects were more obvious. Furthermore, the beams with stirrups exhibited a huge number of cracks with decreased crack spacing but much smaller widths. Moreover, a rise in the (a/d) ratio increased the number of cracks along the beam length, notably in the mid-span region. Shear failure of tested beams occurred on inclined planes with inclination angles around 45°, which was consistent with the method of the truss model for shear analysis. The crack width for beams without NS ranged from 280 μm to 380 μm, whereas the crack width ranged from 121 μm to 198 μm for beams with NS. As the load increased, the shear crack continued to widen, ranging from 1090 μm to 1280 μm for beams without NS and from 1480 μm to 2170 μm for beams with NS. This was due to an increase in tensile concrete strengths because of rising NS. In addition, it can be concluded that the inclusion of NS seemed to have an important effect on the shear crack width of concrete beams reinforced with steel stirrups.

### 3.2. Loads and Vertical Deflections Measured

**Figure 6:**
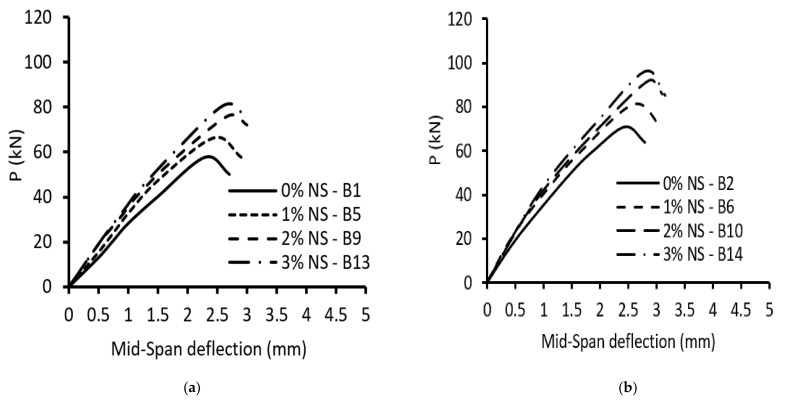
Load–deflection curves of beams with an (a/d) = 1.5: (**a**) without stirrups, ρ**_v_**% = 0%; (**b**) with stirrups, ρ_v_% = 0.38%.

**Figure 7:**
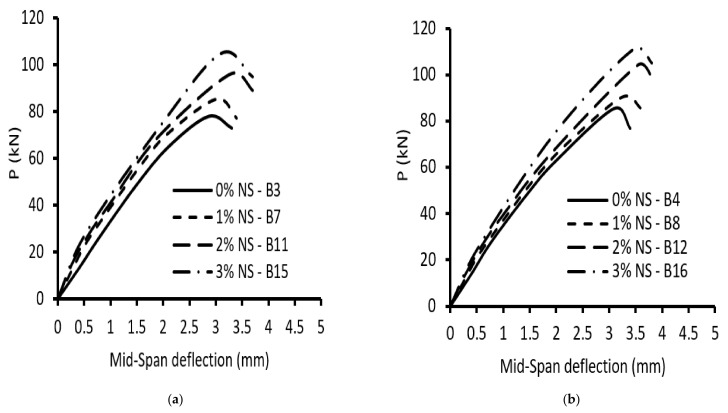
Load–deflection curves of beams with an (a/d) = 2.5: (**a**) without stirrups, ρ**_v_**% = 0%; (**b**) with stirrups, ρ_v_% = 0.38%.

Figure 6 and Figure 7 illustrate the load–deflection curves of the tested beams. The impact of the NS ratio on the ultimate load and maximum mid-span deflection is illustrated in Table 5 and Figure 6 and Figure 7. In general, increasing NS enhanced the load-carrying capacities at all levels, but for the same load level, decreased the vertical deflections at the mid-span. Raising NS from 0 to 1%, 2%, and 3% increased the ultimate load by 13%, 30%, and 39% for specimens without stirrups and an (a/d) of 1.5, respectively, whereas for specimens without stirrups and an (a/d) of 2.5, the ultimate load increased by 15%, 31%, and 36%, respectively. From this, due to the use of NS, the ultimate load increased in roughly the same way for beams with (a/d) of 1.5 and 2.5. The corresponding increase in the mid-span deflection was about 7–15% and 6–16%, respectively. So, the addition of NS to concrete boosted the contribution of concrete to shear strength, as shown by the results of the beams without stirrups. Raising NS from 0% to 1%, 2%, and 3% increased the ultimate load by 8%, 21%, and 30%, respectively, for specimens with stirrups and an (a/d) of 1.5, whereas increasing NS from 0% to 1%, 2%, and 3% increased the ultimate load by 7%, 22%, and 31%, respectively, for specimens with stirrups and an (a/d) of 2.5. Additionally, for specimens with stirrups and an (a/d) of 2.5, the ultimate load increased with approximately the same increase as that in beams with stirrups and an (a/d) of 1.5 due to using NS. The corresponding increases in the mid-span deflection were 8–18% and 9–14%, respectively. Incorporating NS mixture also increased shear fracture resistance, which was due to the importance of NS in concrete’s compressive and tensile strength, which affects shear cracking.

### 3.3. Stirrups Strains

**Figure 8:**
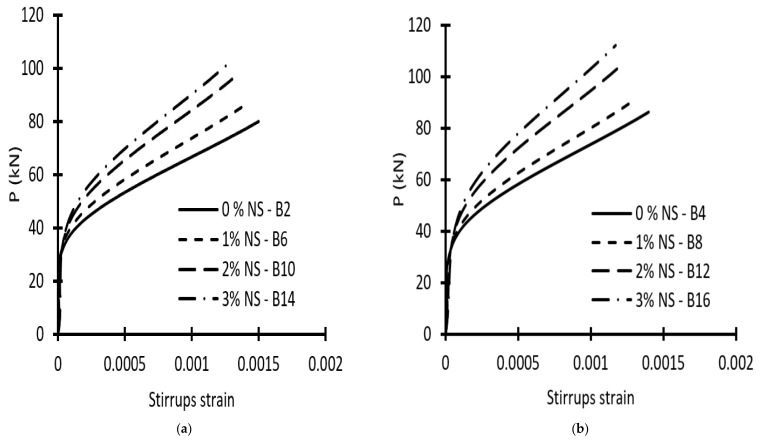
Load-Stirrup strain curves of beams: (**a**) with (a/d) = 1.5; (**b**) with (a/d) = 2.5.

The relations between loads and longitudinal steel strains were approximately linear, since all beams were over-reinforced and the lower longitudinal steel for all specimens was not yielded. Furthermore, the results demonstrated that increasing NS reduced tensile steel strain and concrete compressive strain at various load levels. The load–tensile stirrups strain correlations in the vertical legs of the stirrups passing through the failure plane (about in the middle of the shear span) are shown in Figure 8. The increase in NS resulted in an increase in load-carrying capacity and a decrease in the stirrup strain at various load levels. Concerning specimens with stirrups and an (a/d) of 1.5, increasing NS from 0% to 1%, 2%, and 3% caused a decrease in the stirrups strain by about 16%, 28%, and 33%, respectively, at the failure load. When NS was increased from 0% to 1%, 2%, and 3% for specimens with stirrups and an (a/d) of 2.5, the stirrups strain was reduced by about 14%, 25%, and 27% at the failure load, respectively. The studies also revealed that raising NS from 2% to 3% had a minor influence on stirrups strain.

## 4. Code Provisions

### 4.1. ACI 318-19

The shear critical section was at a distance of d from the column face [16]. The total shear stress (*v_u_*) in a concrete beam reinforced with stirrups was resisted by the concrete (*v_c_*) and the stirrups (*v_s_*).

*v_u_* = *v_c_* + *v_s_* (1)

*v_c_* = 0.17 *λ*(*f_c_*′)^0.5^ b_w_ d (MPa) (2)

*v_s_* = [A_v_
*f_ys_* d/s] (3)

where *λ* is equal to one for normal concrete; *f_c_*′ is the cylinder concrete compressive strength in (MPa); b_w_ is the concrete beam width in mm; d is the effective beam depth in mm, within the spacing, s; A_v_ denotes the area of shear reinforcement; *f_ys_* is the yield strength of shear reinforcement (MPa).

### 4.2. EC 2

The critical section for shear was located at a distance of 2d from the column face [17]. The design value for concrete shear resistance (*v_c_*) was calculated as follows:

*v_c_* = [(0.18/γ_c_) *K* (100 ρ_L_ *f_ck_*)^1/3^] b_w_ d < 0.50 b d ν *f_cd_* (4)

*K* = 1 + (200/d)^1/2^ < 2.0 d in mm (5)

ρ_L_ = A_sl_/(bd) < 0.02 (6)

ν = 0.6 [1 − (*f_ck_*/250)] *f_ck_* in MPa (7)

where *f_ck_* denotes the concrete’s characteristic compressive cylinder strength after 28 days, and *f_cd_* is the concrete compression force design value in the longitudinal beam axis direction. Shear strength resisted by stirrups (*v_s_*) should be taken as the lesser of *v_s_*_,1_ or *v_s_*_,2_:

*v_s_*_,1_ = (A_sw_/S) Z *f_ywd_* (8)

*v_s_*_,2_ = b_w_ Z ν *f_cd_*/(cot θ + tan θ) (9)

where the angle created by the concrete compression struts and the main tension chord is θ, and Z denotes the inner lever arm at the maximum bending moment that is taken as equal to 0.9d. Within the spacing S, A_sw_ is the cross-sectional area of the shear reinforcement. *f_ywd_* is the shear reinforcement’s design yield strength.

### 4.3. ECP 207

The shear critical section is at a distance of d/2 from the column face [18]. The total shear stress (*v_u_*) is calculated as ACI 318-19, except the shear strength resisted by the concrete (*v_c_*), which is calculated as follows:

*v_c_* = 0.24 (*f_cu_*/γ_c_)^0.5^ (10)

where *f_cu_* is the cube concrete compressive strength and γ_c_ is the safety factor for concrete and taken as equal to 1.50.

## 5. Comparison of Test Results with Code Predictions

The envelopes of the experimental shear failure stress (*v_uexp_*) of the tested beams were compared with that estimated from ACI 318-19 (*v_uACI_*), EC-2 (*v_uECP_*), and ECP 207 (*v_uECP_*) codes, as illustrated in Table 6. This comparison demonstrated that the shear strength estimated using the equations of the ACI 318-19 was more conservative than EC-2 and ECP 207 for NS concrete beams. Additionally, Table 6 illustrates that the studied codes are safer in predicting ultimate shear strength for beams with a high NS ratio than for those with a lower NS ratio. In addition, the international studied codes are safer for predicting NS concrete contribution for shear. The average values of the ratios between the experimental shear failure stress and that estimated from ACI 318-19 (*v_uexp_*/*v_uACI_*), EC-2 (*v_uexp_*/*v_uEC_*), and ECP 207 (*v_uexp_*/*v_uECP_*) were 1.64, 1.41, and 1.36, respectively.

## 6. Analysis Using Finite Elements

### 6.1. Modeling Using Finite Elements

The shear strength of the beams with and without NS was evaluated using a 3D nonlinear finite element analysis using the ABAQUAS program [19]. To obtain an appropriate stress distribution in the 3D analysis, the concrete component of the model was split into so-called brick elements using the C3D8R element. The longitudinal and stirrups reinforcement were considered T3D2-embedded truss elements.

### 6.2. Reinforcement

The element T3D2 was used to model beam reinforcement, longitudinal reinforcement, and stirrups; this two-dimensional element is made up of two nodes, each of which has two degrees of freedom. The bottom longitudinal beam reinforcement was specified as four bars assigned with a reinforcement of 254 mm^2^ area for one bar, and the top longitudinal reinforcement was specified as two bars assigned with a reinforcement of 78.50 mm^2^ for one bar. For the beams with stirrups, one stirrup was specified as two legs with an area of 78.5 mm^2^ for one leg and spacing of 166.67 mm. The modulus of elasticity, E_s_, and Poisson’s ratio, were equal to 2 × 10^5^ MPa and 0.30, respectively. The longitudinal and stirrups reinforcements used had yield strengths of 410 MPa and 243 MPa, respectively. There was perfect contact between the concrete and the bar reinforcement.

### 6.3. Concrete

The concrete was modeled with the element C3D8R. Eight nodes, each with three degrees of freedom, define the element, with translation in the x, y, and z dimensions at each node. To mimic concrete behavior, the concrete damage plasticity model was utilized, which defines the compression and tension deterioration of concrete. The damaged property diminishes the elastic stiffness of the element when it plasticizes. The experimental elasticity modulus of concrete was 31,140 MPa, 32,390 MPa, 34,195 MPa, and 34,449 MPa, for concrete with *f_c_*′ = 50.1 MPa, *f_c_*′ = 54.2 MPa, *f_c_*′ = 60.4 MPa, and f_c_′ = 61.3 MPa, respectively. Figure 1 depicts the compressive uniaxial stress–strain values for the ascending and descending regions of the HSC concrete model with and without NS. The failure surface in the concrete damage plasticity model was controlled by ε_t_^−pl^ and ε_c_^−pl^, which are tensile and compressive equivalent plastic strains, respectively. Tensile damage d_t_ and compressive damage d_c_ are two damage variables that describe the loss of elastic stiffness. The damage variables can range from zero to one, with zero representing undamaged concrete and one signifying total loss of strength. Under uniaxial tension, σ_t_, and compression, σ_c_, loading, the stress–strain relationships are [19]:

σ_t_ = (1 − d_t_) E_o_ (ε_t_ − ε_t_^−pl^) (11)

σ_c_ = (1 − d_c_) E_o_ (ε_c_ − ε_c_^−pl^) (12)

where E_o_ is the concrete’s initial elastic stiffness and ε_t_ and ε_c_ are the total tensile and compressive strains, respectively. The expansion angle, ψ, and eccentricity, λ, are the yield surface flow rule parameters that were set to 30° and 0.10, respectively. The yield surface shape was controlled by the parameter K, which is equal to 0.1667. The model’s viscosity parameter was equal to 0.0005 to satisfy the accuracy and convergence.

### 6.4. The Finite Element Mesh

**Figure 9:**
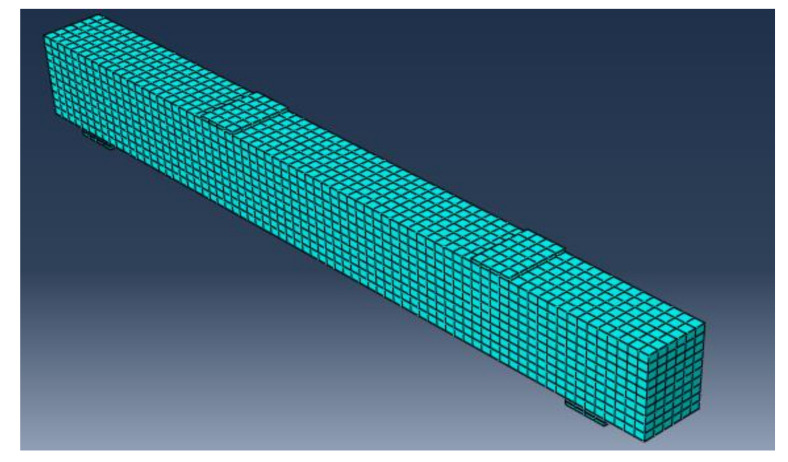
Concrete volume meshes for tested beams.

**Figure 10:**
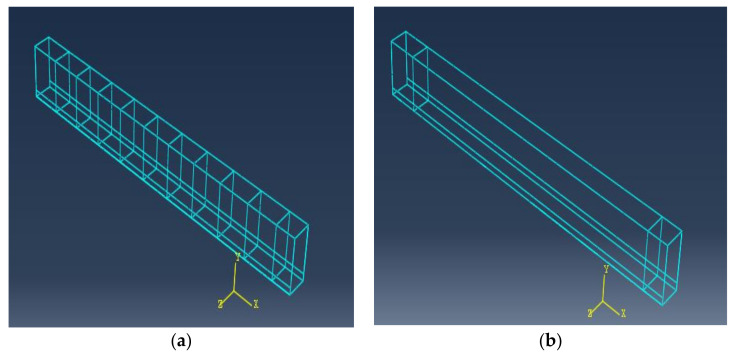
Beam reinforcement meshes: (**a**) with stirrups; (**b**) without stirrups.

All of the elements in the finite element model were purposely assigned the same mesh size to obtain correct results, ensuring that every two different materials share the same node. The 3D solid element C3D8R, size 25 × 25 × 25 mm, was the mesh element for concrete, and the 2D element T3D2, size 25 mm, was the mesh element for longitudinal bars. Four steel plates with dimensions of 100 × 150 × 30 mm were used for supports under the two-point load. Figure 9 and Figure 10 depict concrete volume meshes and reinforcing meshes for some of the specimens that were evaluated, respectively.

### 6.5. Boundary Conditions

**Figure 11:**
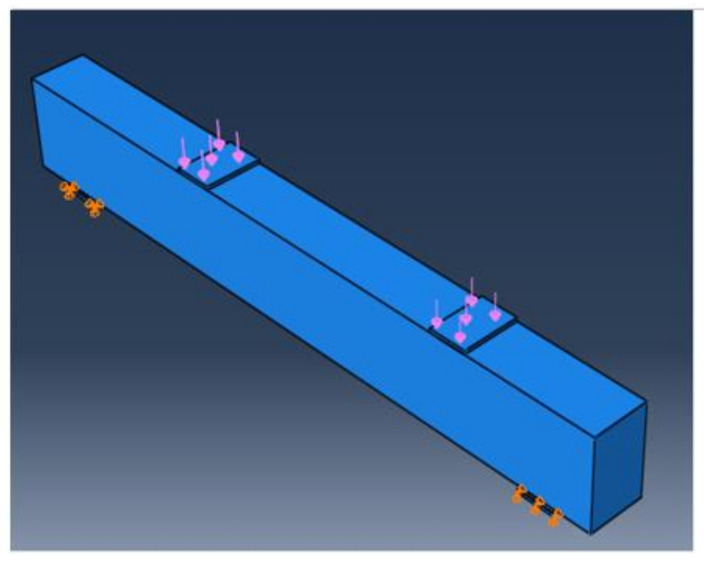
Loading and boundary conditions of analyzed beam.

To simulate the experimental test setup, the model was loaded, and boundary conditions were applied, as illustrated in Figure 11. The right support of the beam was roller support u_1_ ≠ 0.0, u_2_ = u_3_ = 0.0, the left beam support was hinged u_1_ = u_2_ = u_3_ = 0.0, and the two loads were applied at two steel plates located at the top of the beam.

### 6.6. Test Results vs. Finite Element Predictions

**Figure 12:**
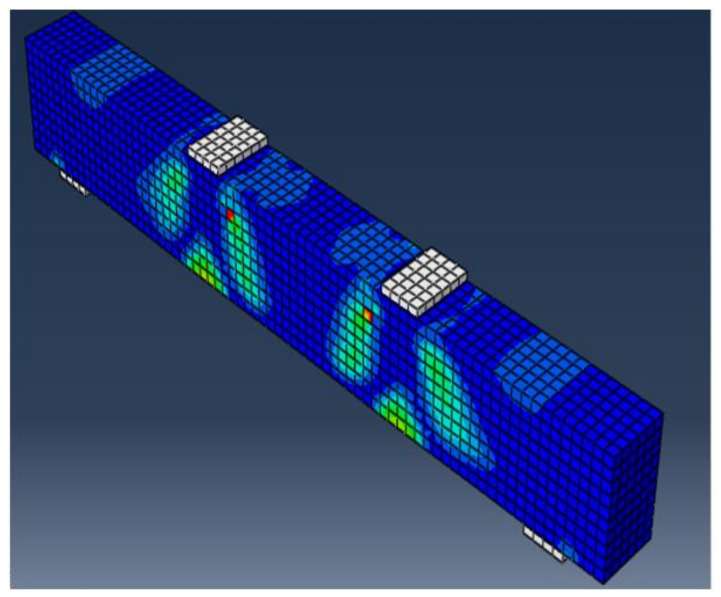
Final crack pattern of specimen B5.

**Figure 13:**
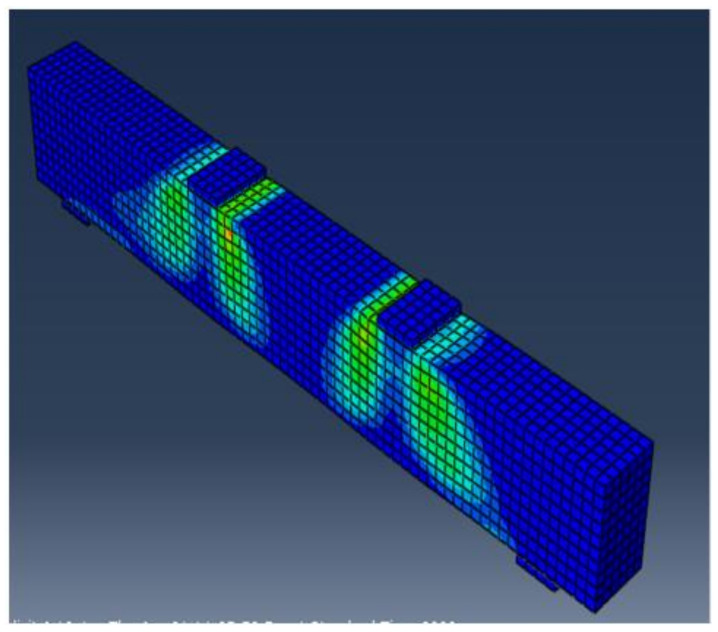
Final crack pattern of specimen B9.

**Figure 14:**
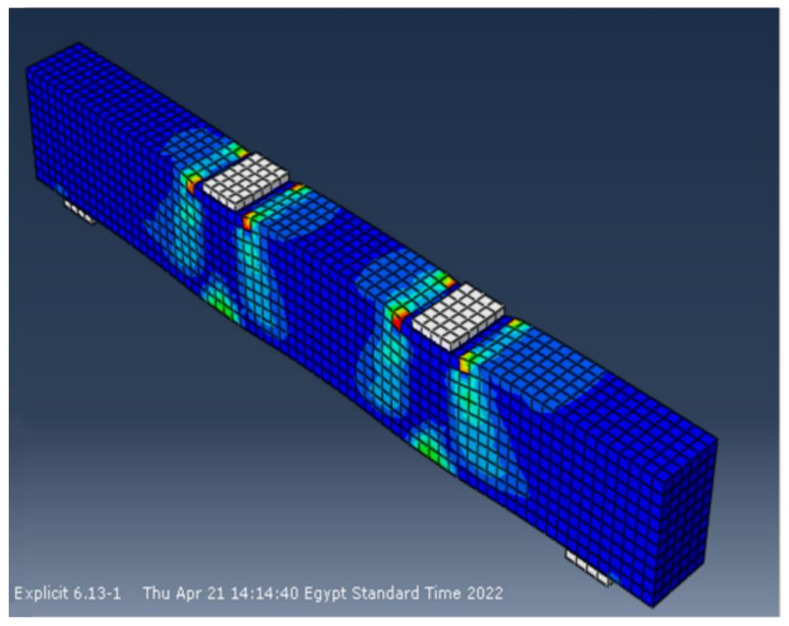
Final crack pattern of specimen B13.

**Figure 15:**
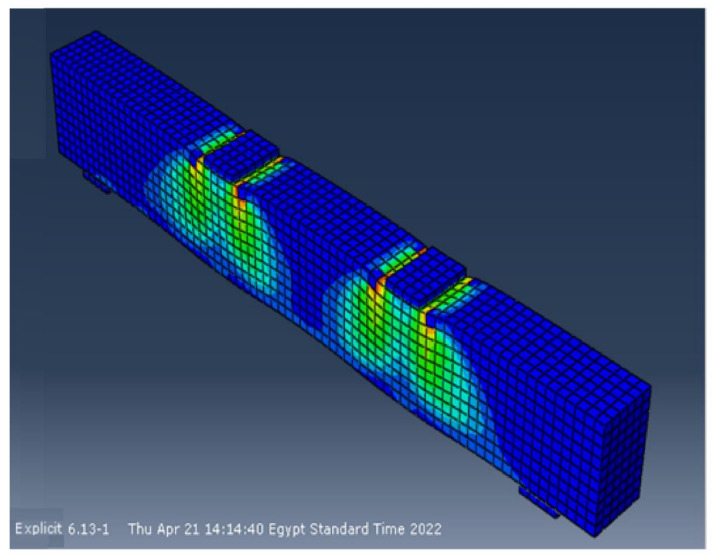
Final crack pattern of specimen B15.

The experimental and finite element crack patterns were found to be very similar. The crack patterns of some of the studied specimens are shown in Figure 12, Figure 13, Figure 14 and Figure 15. Table 6 compares experimental and calculated ultimate load values. The comparison showed that for specimens with and without stirrups and having NS there was a difference of 4–8% between the theoretical and the experimental failure load. Table 7 also compares the maximum displacement measured experimentally at the mid-beam span with the maximum displacement estimated using the finite element method. The comparison shows that, for specimens with and without stirrups and with NS, there was a difference of 5–9% between the theoretical and the experimental mid-span deflection. The foregoing findings demonstrate that the finite element results and experimental measurements were in good agreement. In general, the finite element program ABAQUS can be used to successfully forecast the shear strength of beams with NS, both with and without stirrups.

## 7. Conclusions

Based on the results of the experimental investigations and analytical assessments of the shear behavior of NS beams, the following conclusions can be drawn:

Especially in beams without stirrups, increasing the NS ratio led to fewer cracks, increased crack spacing, and reduced crack width. Additionally, the increasing (a/d) ratio led to an increased number of cracks.

Raising NS from 0% to 1%, 2%, and 3% increased the ultimate load by 13%, 30%, and 39%, respectively, for beams without stirrups and with (a/d) of 1.5, whereas for specimens without stirrups and with (a/d) of 2.5, the ultimate load increased by 15%, 31%, and 36%, respectively.

Raising NS from 0% to 1%, 2%, and 3% increased the ultimate load by 8%, 21%, and 30%, respectively, for beams with stirrups and with (a/d) of 1.5, whereas for beams with stirrups and with (a/d) of 2.5, the ultimate load increased by 7%, 22%, and 31%, respectively.

Raising NS from 0% to 1%, 2%, and 3% increased the mid-span deflection by about 6–18%.

The ACI 318-19 is more conservative than EC-2 and ECP 207 in calculating the shear strength of NS high-strength concrete beams.

The finite element program ABAQUS can be used effectively to predict the shear strength of nano silica-reinforced high-strength concrete beams.

## Figures and Tables

**Table 1:** Nano silica’s properties.

Properties	Description
Specific gravity	1.1–1.30 at 20 °C
Specific surface m^2^/Kg	2 × 10^5^
SiO_2_	>99.50
Solubility	Easily soluble in water
Dynamic viscosity	6–8 MPa
Ph	9.5–10.5
Boiling point	100 °C
Melting Point	0 °C
Flash Point	Inflammable
Pressure of vapor	32 hPa at 25 °C

**Table 2:** Mix proportions.

	NS %	Cement kg/m^3^	NS kg/m^3^	SP %	SP kg/m^3^	Sand kg/m^3^	Crushed Limestone kg/m^3^	Water kg/m^3^	Water/Cement Ratio	Average *f_c_*′ (MPa)	Average *f_cu_* (MPa)	Average *f_t_* (MPa)
1	0	800	0	2	16.00	800	1600	200	0.25	50.10	41.20	3.20
2	1	792	8	2	15.84	800	1600	198	0.25	54.20	44.50	3.51
3	2	784	16	2	15.68	800	1600	196	0.25	60.40	49.60	3.80
4	3	768	32	2	15.36	800	1600	192	0.25	61.30	50.80	3.90

**Table 3:** Slump air content and density of concrete mixtures.

Mix.	Test		
	Slump (mm)	Air Content (%)	Density (kg/m^3^)
Control with 0% NS	192	1.40	2460.2
1% NS	174	0.93	2468.2
2% NS	169	0.83	2469.1
3% NS	169	0.83	2469.1

**Table 4:** Tested beams description.

Serial	Beam Designation	Mix. No.	b mm	t mm	d mm	A_s_	ρ_L_%	A_s_’	a/d	Stirrups ϕ Bar Diameter mm @ Spacing (mm)	ρ_v_%
1	B1	1	150	250	192	4 ϕ 18	3.53	2 ϕ 12	1.5	0	0.00
2	B2								1.5	ϕ 6 @ 100	0.38
3	B3								2.5	0	0.00
4	B4								2.5	ϕ 6 @ 100	0.38
5	B5	2	150	250	192	4 ϕ 18	3.53	2 ϕ 12	1.5	0	0.00
6	B6								1.5	ϕ 6 @ 100	0.38
7	B7								2.5	0	0.00
8	B8								2.5	ϕ 6 @ 100	0.38
9	B9	3	150	250	192	4 ϕ 18	3.53	2 ϕ 12	1.5	0	0.00
10	B10								1.5	ϕ 6 @ 100	0.38
11	B11								2.5	0	0.00
12	B12								2.5	ϕ 6 @ 100	0.38
13	B13	4	150	250	192	4 ϕ 18	3.53	2 ϕ 12	1.5	0	0.00
14	B14								1.5	ϕ 6 @ 100	0.38
15	B15								2.5	0	0.00
16	B16								2.5	ϕ 6 @ 100	0.38

**Table 5:** Experimental results.

Serial	Beam Designation	Mix. No.	a/d	Stirrups ϕ Bar Diameter mm @ Spacing (mm)	ρ_v_%	P_cr_ (kN)	P_u_ (kN)	∆_u_ (mm)	P_cr_/P_u_
1	B1	1	1.5	0	0.00	42.07	58.94	2.29	0.71
2	B2		1.5	ϕ 6 @ 100	0.38	51.10	78.47	2.86	0.65
3	B3		2.5	0	0.00	49.84	70.87	2.45	0.70
4	B4		2.5	ϕ 6 @ 100	0.38	54.67	85.61	3.10	0.64
5	B5	2	1.5	0	0.00	48.51	66.57	2.44	0.73
6	B6		1.5	ϕ 6 @ 100	0.38	53.34	85.12	3.30	0.63
7	B7		2.5	0	0.00	59.92	81.41	2.60	0.74
8	B8		2.5	ϕ 6 @ 100	0.38	66.15	90.58	3.26	0.73
9	B9	3	1.5	0	0.00	54.67	77.21	2.63	0.71
10	B10		1.5	ϕ 6 @ 100	0.38	64.82	96.04	3.08	0.67
11	B11		2.5	0	0.00	63.77	92.68	2.79	0.69
12	B12		2.5	ϕ 6 @ 100	0.38	81.48	104.23	3.46	0.78
13	B13	4	1.5	0	0.00	56.14	81.97	2.68	0.69
14	B14		1.5	ϕ 6 @ 100	0.38	69.44	102.62	3.28	0.68
15	B15		2.5	0	0.00	66.78	96.25	2.85	0.69
16	B16		2.5	ϕ 6 @ 100	0.38	84.35	111.16	3.53	0.76

**Table 6:** Comparison of experimental test results with analyzed codes’ design equations.

Beam Designation	*v_uexp._* (MPa)	*v_uACI_* (MPa)	*v_uEC_* (MPa)	*v_uECP_* (MPa)	*v_uexp_*/*v_uACI_*	*v_uexp_*/*v_uEC_*	*v_uexp_*/*v_uECP_*
B1	2.05	1.20	1.46	1.52	1.71	1.40	1.35
B2	2.78	2.73	2.79	3.05	1.02	1.00	0.91
B3	2.46	1.20	1.46	1.52	2.05	1.68	1.62
B4	2.97	2.73	2.99	3.05	1.09	0.99	0.97
B5	2.31	1.25	1.52	1.58	1.85	1.52	1.46
B6	2.96	2.78	3.05	3.11	1.06	0.97	0.95
B7	2.83	1.25	1.52	1.58	2.26	1.87	1.79
B8	3.15	2.78	3.05	3.11	1.13	1.03	1.01
B9	2.68	1.32	1.60	1.67	2.03	1.68	1.60
B10	3.33	2.85	3.13	3.20	1.17	1.07	1.04
B11	3.22	1.32	1.60	1.67	2.44	2.02	1.93
B12	3.62	2.85	3.13	3.20	1.27	1.16	1.13
B13	2.85	1.33	1.61	1.68	2.14	1.78	1.70
B14	3.56	2.86	3.14	3.21	1.24	1.14	1.11
B15	3.34	1.33	1.61	1.68	2.51	2.08	1.99
B16	3.86	2.86	3.14	3.21	1.35	1.23	1.20
Average value					1.64	1.41	1.36

**Table 7:** Comparison between the experimental and finite element results.

Beam Designation	Failure Load (kN)		Max. Mid-Span Deflection (mm)	
	Finite Element	EXP./Finite Element	Finite Element	EXP./Finite Element
B1	62.91	0.94	2.56	0.89
B2	83.12	0.94	3.15	0.91
B3	72.28	0.98	2.72	0.90
B4	91.41	0.94	3.39	0.91
B5	70.87	0.94	2.71	0.90
B6	89.97	0.95	3.60	0.92
B7	86.21	0.94	2.88	0.90
B8	95.61	0.95	3.56	0.92
B9	81.75	0.94	2.91	0.90
B10	101.32	0.95	3.37	0.91
B11	94.65	0.98	3.07	0.91
B12	106.65	0.98	3.76	0.92
B13	82.73	0.99	2.96	0.91
B14	104.8	0.98	3.58	0.92
B15	102.56	0.94	3.14	0.91
B16	116.96	0.95	3.84	0.92

## Data Availability

All the data supporting reported results can be found in the manuscript.
